# Widespread chromosomal rearrangements preceded genetic divergence in a monitor lizard, *Varanus acanthurus* (Varanidae)

**DOI:** 10.1007/s10577-023-09715-x

**Published:** 2023-02-06

**Authors:** Jason Dobry, Erik Wapstra, Emily J. Stringer, Bernd Gruber, Janine E. Deakin, Tariq Ezaz

**Affiliations:** 1 Centre for Conservation Ecology and Genomics, Institute for Applied Ecology, University of Canberra, Canberra, ACT 2617 Australia; 2 School of Natural Sciences, University of Tasmania, Hobart, Tas 7001 Australia

**Keywords:** Chromosomics, Speciation, Adaptation, Drift, Population genetics, Cytogenetics

## Abstract

Chromosomal rearrangements are often associated with local adaptation and speciation because they suppress recombination, and as a result, rearrangements have been implicated in disrupting gene flow. Although there is strong evidence to suggest that chromosome rearrangements are a factor in genetic isolation of divergent populations, the underlying mechanism remains elusive. Here, we applied an integrative cytogenetics and genomics approach testing whether chromosomal rearrangements are the initial process, or a consequence, of population divergence in the dwarf goanna, *Varanus acanthurus*. Specifically, we tested whether chromosome rearrangements are indicators of genetic barriers that can be used to identify divergent populations by looking at gene flow within and between populations with rearrangements. We found that gene flow was present between individuals with chromosome rearrangements within populations, but there was no gene flow between populations that had similar chromosome rearrangements. Moreover, we identified a correlation between reduced genetic variation in populations with a higher frequency of homozygous submetacentric individuals. These findings suggest that chromosomal rearrangements were widespread prior to divergence, and because we found populations with higher frequencies of submetacentric chromosomes were associated with lower genetic diversity, this could indicate that polymorphisms within populations are early indicators of genetic drift.

## Introduction

One of the central debates in species evolution, characterized by both evolution within species and evolution between species, is whether chromosomal rearrangements initiate divergence between populations or result from the speciation process (King 1993; Livingstone and Rieseberg 2003; Potter et al. 2017; Deakin 2018; Damas et al. 2021). There are many studies over the last century investigating the phenomena of chromosomal rearrangements and their relationship to the speciation process by disrupting gene flow. These rearrangements result in the deletion, insertion, duplication, or inversion of DNA sequences and alter the recombinant framework of allelic structure within chromosomes (White 1969; King 1993; Faria and Navarro 2010; Potter et al. 2017). The first example that demonstrated chromosomal rearrangements as potential drivers of speciation came from seminal work on the vinegar fly (*Drosophila melanogaster*), when Alfred Sturtevant crossed a wild female to a male with mutations on the second chromosome. He observed that when a F1 female was backcrossed, there was no recombination in any offspring for the mutant alleles that had previously had a crossover frequency of 37% (Sturtevant 1917). This observation subsequently led to the use of chromosome rearrangements as an indicator of potential genetic isolation, and they were thus the first genetic markers used for constructing phylogenetic maps demonstrating genetic distances between species (Sturtevant 1921). More recent work in *D. melanogaster* showed that fixed inversions were associated with phenotypic variation such as size differences in the wings and thorax, thermal tolerance, longevity, and diet requirements in wild populations (Hoffmann and Riesberg 2008; Kirkpatrick 2010; Fuller et al. 2019; Zivanovic et al. 2021). Beyond *Drosophila*, studies exploring the role of chromosome rearrangements on a wide range of taxa from *Saccharomyces cerevisiae* (Lu and He 2019) and many species of plants (Huang and Rieseberg 2020) to chimpanzees and humans (Locke et al. 2003; Szamalek et al. 2007) have revealed that in most cases, chromosomal rearrangements were fixed differences between groups and gene flow was restricted. Chromosome polymorphisms are well studied in *Drosophila* and other invertebrates including *Papilio* and *Heliconius* butterflies, bees, ants, and many plants (Kronforst et al. 2013; Gallant et al. 2014; Iijima et al. 2018; Wellenreuther and Bernatchez 2018; Huang and Rieseberg 2020). However, chromosome polymorphisms within species in natural populations of vertebrates are far less common and believed to be temporary transitions during chromosomal evolution (Farré et al. 2016; Damas et al. 2021, 2022). Typically, selection drives rearrangements to fixation (or extinction), which has been repeatedly demonstrated throughout vertebrate evolution (Damas et al. 2021; Deakin and Ezaz 2014; 2019; Sacerdot et al. 2018). However, it remains unclear if chromosomal rearrangements are the primary driver that leads to the restriction of gene flow or if they are secondary to other drivers of divergence between groups.

**Fig. 1:**
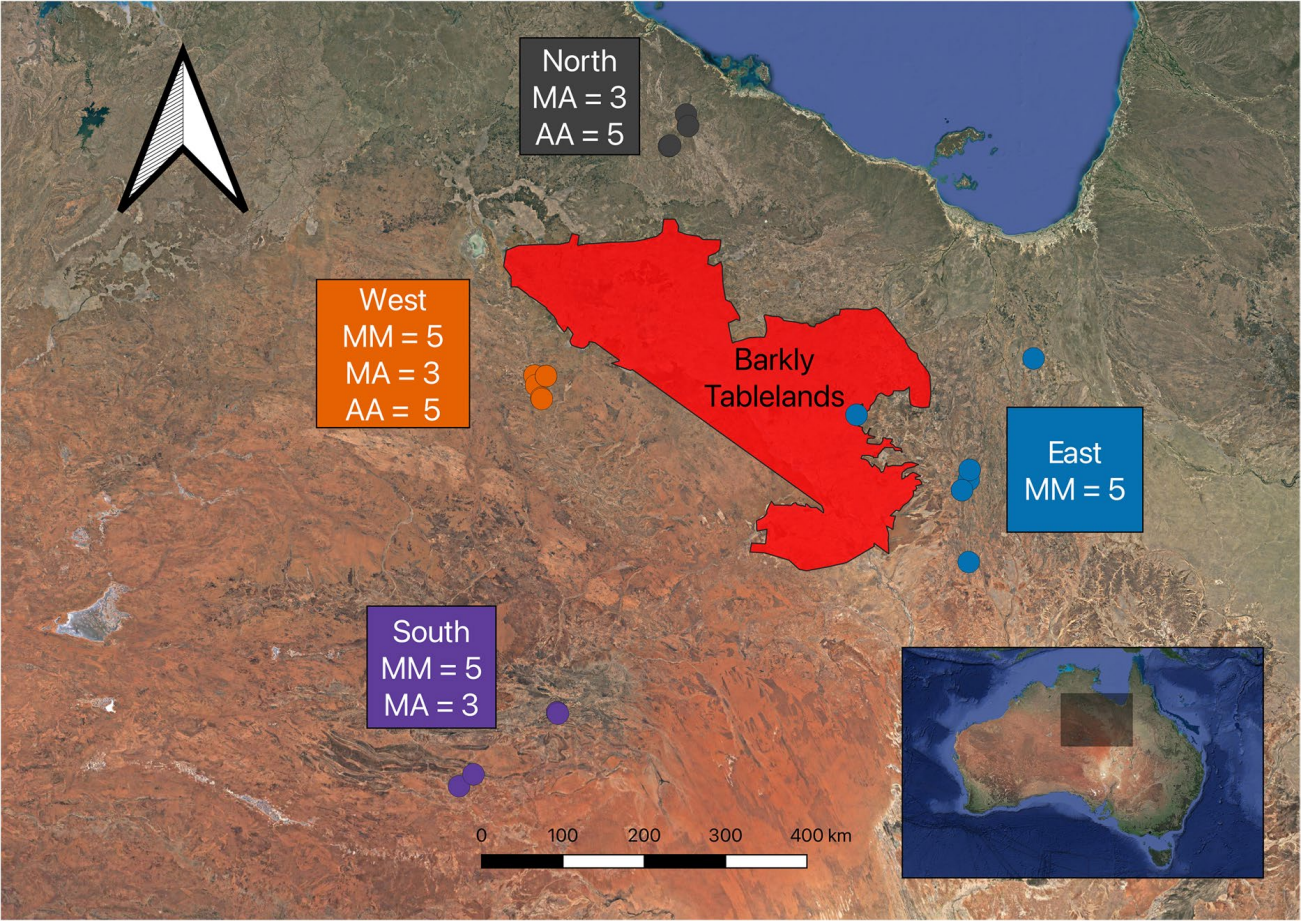
Sampling localities of *Varanus acanthurus* populations on north, south, east, and west of the Barkly Tablelands (red polygon). Each population was karyotyped and genotyped to compare both genetic and chromosomal divergence between and within each population. Individual karyotypes for polymorphisms on chromosome six are indicated for each population (homozygous submetacentric (MM), heterozygous submetacentric acrocentric (MA), and homozygous acrocentric (AA)). The east is monomorphic for chromosome 6, the north had two morphologies (AA and MA), the west had all three morphologies (MM, MA, and AA), and the south had two morphologies (MM and MA)

Varanidae, a widespread family of reptiles found in Africa, the Middle East, Asia, and Australia, provides an ideal model system for testing the role of chromosome rearrangements in speciation. Varanids are distinct from many other species of lizards in that they have a remarkably conserved karyotype where all species so far karyotyped have 2n = 40 chromosomes (King and King 1975; Dutt 1968; King et al. 1982; De Smet 1981; Matsubara et al. 2014; Patawang et al. 2017; Johnson Pokorná et al. 2016; Iannucci et al. 2019; Augstenová et al. 2021). All variability in their karyotypes is due to differences in chromosome morphology on chromosomes 3–8. The morphological differences are presumably all due to inversions or centromere repositioning and in most cases are fixed differences between species. There is, however, one known exception, a dwarf species from northern Australia, the ridge-tailed goanna (*Varanus acanthurus*), which is unique among varanids in having within-species chromosome polymorphisms (King et al. 1982; Matsubara et al. 2014). In this widespread species, distributed from the coastline in Western Australia to both arid and tropical regions of central Queensland, chromosome polymorphisms characterized as pericentric inversions have been reported between two races on either side of the Barkly Tablelands in central Australia (King et al. 1982) (Fig. 1). The populations on the east side of the Barkly Tablelands are homozygous for submetacentric chromosome 6 (MM:6), while the western race had individuals that were either homozygous for an acrocentric chromosome 6 (AA:6), homozygous submetacentric (MM:6), or heterozygous submetacentric acrocentric (MA:6). This observation is intriguing because the chromosome positioning of centromeres are generally highly stable on an evolutionary time scale (Lu and He 2019). This stability of centromere positioning was demonstrated with fission yeast (*Schizosaccharomyces pombe*) in which progeny has shown severe lethality due to failure in meiotic chromosome malsegregation (Lu and He 2019). A recent review on inversions (Faria et al. 2019) indicated that when a chromosome mutation results in an inversion polymorphism, the result (assuming positive or neutral selection) is a derived chromosome with a single new sequence arrangement. During cell division in the germline, this newly derived chromosome becomes isolated from the inherent genetic variation established during normal recombination events within the collinear sequence (Faria et al. 2019). The only way for genetic variance to enter a newly rearranged chromosome segment is from rare events of mutation or gene flux between the inverted and ancestral arrangement. The derived chromosome morphology can only evolve into a divergent collinear genome with frequent recombination following the fixation of this derived morphology in the population and then exchanging the accumulated mutations during its period of isolation.

In this study, we applied a chromosomics (Claussen 2005; Deakin et al. 2019) approach that integrated the use of molecular cytogenetics and high-throughput genotyping by sequencing (GBS) technology in *Varanus acanthurus*. The study included individuals collected from four sites, to the north, south, east, and west of the Barkly Tablelands (Fig. 1), a region roughly 100,000 km^2^ dominated by Mitchell grass (*Astrebla pectinata*) situated between Tennant Creek in the Northern Territory and Mount Isa in Queensland (Williams 1928). Although this grassland is known for seasonal or periodic monsoonal flooding, it was not considered to be a likely geographical barrier for these active lizards, and a genetic barrier was hypothesized as the explanation for the two chromosomal races (King et al. 1982). We applied this approach to address the overarching question asking do chromosome rearrangements initiate genetic divergence or result from it? There are two possible scenarios, the first is that chromosome rearrangements are a result of slow genic changes associated with population divergence and secondary contact from divergent populations. In this situation, we expect an abrupt sink of gene flow in narrow hybridization zones associated with genetic structure between karyotype differences similar to what has been shown in *Sceloporus* lizards (Bedoya and Leaché 2021). The second possibility is that rearrangements have preceded divergence as hypothesized in the split between *Drosophila persimilis* and *D. pseudoobscura* where chromosomal inversions were potentially already present in their ancestral population prior to the species split (Fuller et al. 2018). In the latter situation, gene flow between karyotypes would only be prevented within the rearrangement, leaving a heterogeneous pattern of divergence across the genome and resulting in “speciation with gene flow.” Specifically, we tested the following approach: (i) can populations be identified by chromosomal morphology with cytogenetic analysis alone and (ii) do those chromosomal morphologies correlate with patterns of gene flow either within or between populations using population genetic analysis? This combined approach which integrated a population genetics tool with cytogenetic analysis allowed us to test the temporal question of which evolutionary indicator (rearrangement or divergence) came first by identifying the directionality of gene flow patterns (or lack thereof) within populations and between subpopulations by observing allele frequencies of private and fixed alleles and how they correlate with chromosome morphology in divergent populations. We discuss the implications of our findings in the context of chromosome rearrangements initiating genetic divergence independent from genetic barriers and population divergence.

## Materials and methods

### Sample collections

We collected individuals from locations on four sides of the Barkly Tablelands (Fig. 1). A summary of the number of individuals sampled per location is indicated in Table 1. In the west, we collected 10 individuals, and two of the females maintained in the laboratory for cytogenetic analysis laid eggs shortly, thereafter increasing our sample size from that population to 21. In the north and south populations, we collected nine individuals each and karyotyped eight of them (in one individual from each of these two populations, we were unable to culture leucocytes or establish cell lines). In the east, we collected five individuals and supplemented this population with five museum specimens (Table 1). The museum specimens were collected within the range of the homozygous submetacentric race as described by King et al. (1982), and those specimens are inferred as submetacentric during bioinformatic analysis.

**Table 1:** Summary of *Varanus acanthurus* collection data and the number of individuals for downstream analysis from four sites around the Barkly Tablelands

# individuals	Locality	Sex (M.F)	# karyotyped	# genotyped
9	North	4.4	8	9
9	South	6.2	8	9
10	East	5.0	5	10
21	West	6.7	13	21

On capture, blood samples were taken from the caudal vein and were stored in a proteinase K cell lysis buffer (Sambrook and Russell 2001) which was modified to a concentration of 50 mM EDTA for field collection. Individuals for karyotype analysis were transported to the University of Canberra and maintained in terraria as described by Retes and Bennett (2001). Cell cultures were established from both blood cultures (King and King 1975; Pokorná et al. 2010) and primary cell lines. Cell growth was inconsistent with blood cultures as has been described previously (King and King 1975), and these were only used for individuals that cell lines could not be established from tail tissues (Ezaz et al. 2008).

### Karyotype analysis

A total of 34 individuals were karyotyped (Fig. 1 and Table 1). Fibroblast cells were cultured and propagated using the methods described previously (Ezaz et al. 2008) with slight modifications. Briefly, animals were thoroughly washed with chlorhexidine soap, and the tail tips were removed with a sterile scalpel and further cleaned to remove any old scales, and then the tail tips were soaked in a 6% (v/v) hydrogen peroxide bath for 5 min followed by a betadine wash and then immediately transferred to Hanks Balanced Salt Solution (Sigma Aldrich) with 1 × antibiotic–antimycotic (Thermo Fisher Scientific Australia Pty Ltd., Scoresby, Victoria, Australia) and allowed to macerate overnight at 28 °C. The tail tips were then washed in phosphate-buffered saline (PBS), and the scales were removed with a scalpel, and the tissues were finely diced in PBS buffer and then transferred to T25 culture flasks with Amniomax C-100 basal media and supplement (Gibco, Thermo Fisher Scientific Australia Pty Ltd.) with 1 × antibiotic–antimycotic (Thermo Fisher Scientific Australia Pty Ltd.) and incubated at 28 °C with 5% CO_2_. Each tail tip was split into four flasks and monitored daily for signs of cell division. After 10 days, the cells were rapidly dividing, and the most vigorously growing lines were transferred to T75 flasks until they reached 80% confluency. Cells were then harvested and prepared as described by Ezaz et al. (2005). Slides were visualized with a Zeiss Axio Scope A1 epifluorescence microscope equipped with an AxioCam MRm Rev. 3 (Carl Zeiss Ltd., Cambridge, UK) camera, and Metasystems Isis FISH Imaging System V 5.5.10 (Metasystems, Newton, MA, USA) software was used for analyzing and karyotyping the photographs.

### SNP analysis

We used Diversity Arrays Technology (DArT, Bruce, ACT, Australia) for genotyping 49 individuals including the 34 individuals that were karyotyped (Table 1). DArT is a genome-wide SNP typing technology that utilizes complexity reduction and Illumina sequencing (Kilian et al. 2012). DNA samples were digested with restriction enzymes to ~ 500 bp size fragments; these fragments were cloned, amplified, and sequenced using Illumina. The target libraries were generated from all individuals, and the allele differences for each sequence were characterized by 0, 1, or 2 (homozygous, heterozygous, homozygous for opposite allele). These data were compared to the reference clone from Illumina Barcoding (Kilian et al. 2012).

### Data sorting and analysis

The data was analyzed using the dartR package, which was developed specifically for Diversity Arrays Technology output (Gruber et al. 2018). Briefly, the data were initially filtered for call rate by locus and individuals separately with a threshold of 0.8 and 0.75, respectively. Following call rate filtering, we filtered by reproducibility at a threshold of 0.99 and removed monomorphic loci from the whole dataset for initial analysis. We then performed a scree plot of eigenvalues that indicated informative axes (Unmack et al. 2019) and performed principal coordinate analysis (PCA) (Pearson 1901; Hotelling 1933; Jollife 2002; Jollife and Cadima 2016) with no a priori population assignments (Fig. 4a and b). Fixed differences and *F*_st_ analysis were performed for each population to further characterize the distribution and frequencies of alleles between populations (Table 4). To analyze each population independently, we filtered additional monomorphic loci that were population specific. We then used PCA to assess genetic distances for each individual within populations from each locality and assign the karyotypes to individual genotypes within the PCA to observe the distribution of karyotype morphologies with Euclidean genetic distance measures (Fig. 3a, c, e, and g). This allowed for within-population genetic distance measures between individuals with different karyotype morphologies.

To determine gene flow between individuals with different karyotype morphologies within populations, we used both PCA and isolation by distance using a dissimilar measure of unshared alleles (1, proportion of shared alleles). We assigned karyotypes to each individual and plotted the analyses (Fig. 3b, d, f, and h). In Fig. 3, we also used a neighbor-joining tree to demonstrate the relationships between populations based on SNP data and karyotypes.

To investigate genetic differentiation and estimate gene flow between populations, we performed an isolation-by-distance analysis based on the mantel test (Rousset 1997). First, we analyzed each population individually as defined by locality and PCA ordination (north, south, east, and west) pairwise with unshared alleles (1, proportion of shared alleles) for each individual versus distance (Fig. 4c). Next, we analyzed these four populations pairwise for *F*_st_ values (Fig. 4d) versus distance. After testing for isolation by distance, we performed a multiple linear regression model that included genetic distance and Euclidean distance along with an additional term that distinguished between individuals with the same karyotype and individuals with different karyotypes. This allowed us to establish a null hypothesis and test if karyotypes caused a significant difference in isolation-by-distance patterns. We tested this at two spatial scales, firstly for individuals within 50 km of each other for within-population comparison and secondly for the whole extent of the data, individuals up to 1030 km apart, for the between-population comparison.

## Results

### Karyotypes in Varanus acanthurus

**Fig. 2:**
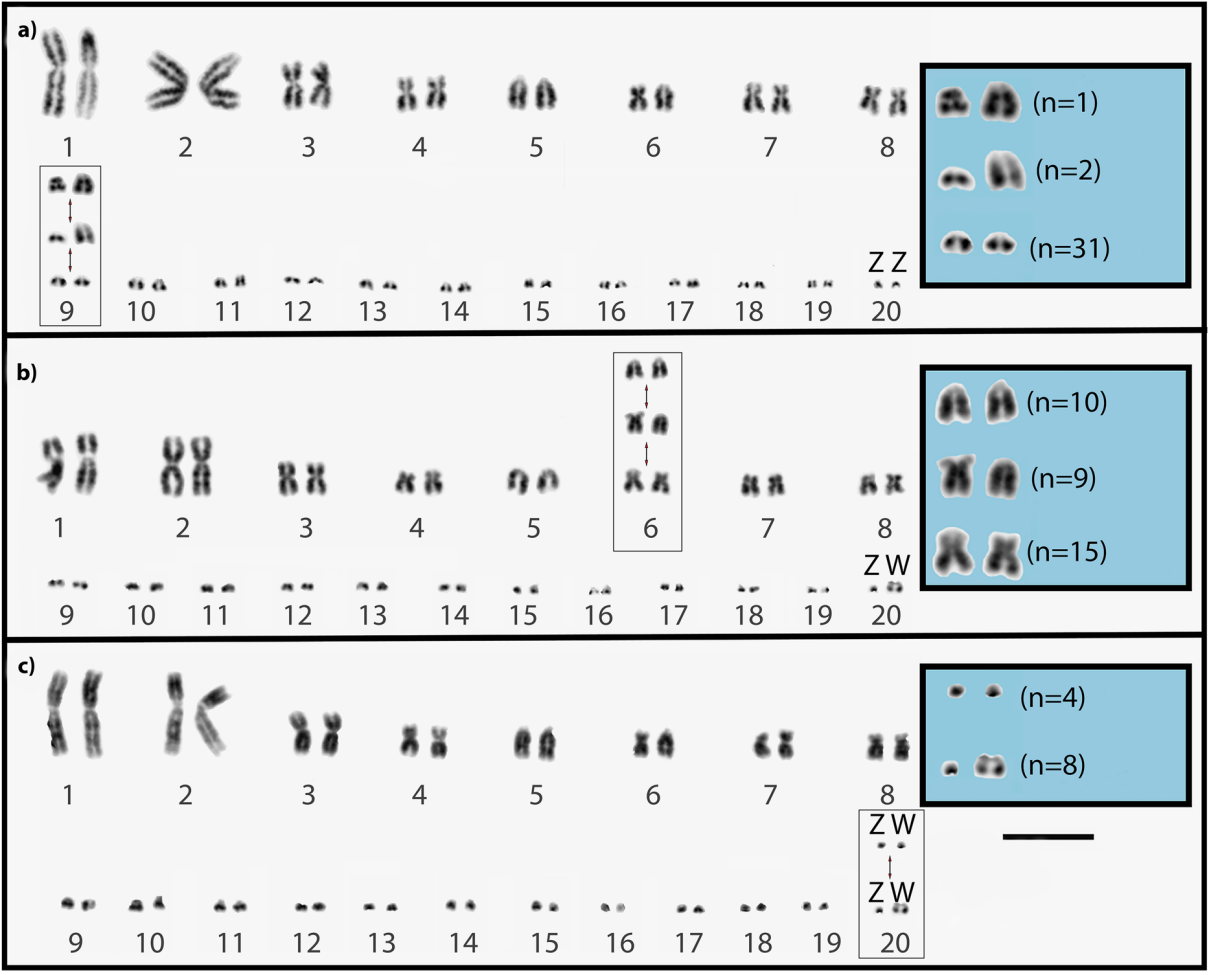
*Varanus acanthurus* karyotype polymorphisms for chromosome pair 6, 9, and 20 (sex chromosomes). **a** Enlarged microchromosomes (pair 9) seen in three individuals. **b** Polymorphic variation observed on chromosome 6. **c** Sex chromosome variation identified in the north population only (pair 20). Scale bar represents 5 µm

All 34 karyotyped individuals had a conserved karyotype number of 2n = 40 with 16 macrochromosomes and 24 microchromosomes as described previously (Augstenová et al. 2021; King et al. 1982; King and King 1975; Iannucci et al. 2019; Srikulnath et al. 2013; Matsubara et al. 2014; Patawang et al. 2017; De Smet 1981). However, we observed multiple morphological chromosomal polymorphisms within and between our four populations (summarized in Table 2). Polymorphisms included enlarged acrocentric microchromosomes (chromosome 9) (Fig. 2a), polymorphisms with three morphologies associated with one macrochromosome (chromosome 6) (Fig. 2b), and polymorphisms involving ZW sex chromosomes (Fig. 2c). Only the northern population had multiple polymorphisms in the same individual. (One female had enlarged autosomal microchromosomes and homomorphic sex chromosomes and had MA:6 karyotype). The other females from that population all had homomorphic sex chromosomes and had either AA:6 or MA:6 and did not have enlarged microchromosomes).

**Table 2:** Karyotype morphology and polymorphism distribution for each locality of *Varanus acanthurus*. A total of 34 individuals were karyotyped; 8 each from the north and south, 5 from the east, and 13 from the west of the Barkly Tablelands

Locality	Chromosome 6			Microchromosomes		Sex Chromosomes			
				Enlarged unpaired	Enlarged paired	Heteromorphic		Homomorphic	
	MM	MA	AA			Males	Females	Males	Females
North	0	3	5	0	1	0	0	4	4
South	5	3	0	0	0	0	2	6	0
East	5	0	0	2	0	0	0	5	0
West	5	3	5	0	0	0	7	6	0

### Polymorphisms in macrochromosome 6

We observed polymorphisms at chromosome 6 from both males and females in three populations (north, west, and south; see Fig. 2b). The fourth population (east) was fixed for the submetacentric morphology. The overall karyotype frequencies for chromosome 6 were 44% MM:6 (15/34 individuals), 29% AA:6 (10/34 individuals), and 26% heterozygous MA:6 (9/34 individuals).

Each locality had a unique distribution and frequency of karyotypes. In the north, 62.5% (five out of eight individuals; three males and two females) were AA:6, and 37.5% (two females and one male) were heterozygous (MA:6), and there were no individuals with the homozygous submetacentric (MM:6) morphology. In the south, we found the opposite, 62.5% of individuals were MM:6, and the remaining 37.5% were heterozygous (MA:6), and there were no individuals with the AA:6 morphology. In the west, there was an even distribution of the two homozygous karyotypes; AA:6 individuals (38.5%) and MM:6 individuals (38.5%) and only 23% were heterozygous. In the east, all five individuals karyotyped had a MM:6 karyotype.

### Polymorphisms involving microchromosomes

We observed enlarged microchromosomes in only three out of 34 individuals from two localities (east and north). In the north, 12.5% (one of eight) karyotyped individuals had an enlarged acrocentric microchromosomes pair 9 (Fig. 2a). In the east, 40% (2 of 5) karyotyped individuals had an unpaired enlarged acrocentric microchromosome pair 9 (Fig. 2a). We did not observe any enlarged microchromosomes that were unrelated to the sex chromosomes in the west or south populations (Fig. 2a).

### Polymorphisms involving sex chromosomes

We observed size variation in the sex chromosomes in the north (Fig. 2c). All eight karyotyped individuals (four males and four females) from this population had homomorphic sex chromosomes (Fig. 2c). In the east population, we only collected males, so we did not have morphological data for the W chromosome from that population. All the females from the west and south (*n* = 9) had enlarged W chromosomes.

### Population genetic analysis using SNPs

The unfiltered DArTseq dataset contained 301,738 SNP loci (Table 3). These data were filtered for analysis, and the resulting dataset contained 46,189 loci (Table 3). Due to the high divergence between populations and the many fixed (monomorphic) alleles that were population specific, we filtered each population individually for monomorphic loci (see Table 3).

**Table 3:** SNP loci before and after filtering for genetic distance measures. The individual populations required additional filtering for monomorphic loci resulting in the post-filtering allele values for each population

Dataset	Number of individuals	Number of SNP alleles		Number of monomorphs
		Pre-filtering	Post-filtering	
All populations	49 (M + F)	301,738	46,189	195,906
North	9 (M + F)	46,189	19,039	26,504
West	21 (M + F)	46,189	17,075	29,114
East	10 (M + F)	46,189	14,908	31,281
South	9 (M + F)	46,189	7733	38,439

### Genetic structure within populations

To detect structure within populations, we analyzed the populations independently. Each population had a significant number of unique monomorphic loci, private alleles, and fixed differences (Tables 4 and 5), and each population had chromosome polymorphisms. Private alleles are defined as cases where one population has a private allele (PA) compared to another population, but the reverse is not true. Fixed differences (FD) are cases where both populations are homozygous for the opposite allele, and monomorphic loci (M) are loci that are fixed at both alleles for any given population(s). In some cases, an allele can be both M in one population and PA in another population; this is because the PA is a heterozygote in the first population and a homozygote for one of the alleles in the M population.

**Table 4:** Pairwise comparison of private alleles and fixed differences between populations. Populations, the number of individuals per population, the number of loci with fixed allele differences between each population, the private alleles for each population, the total private alleles for each pairwise comparison between populations and the Fst between populations

pop1	pop2	N1	N2	Fixed	priv1	priv2	Total priv	Fst
East	North	10	9	4076	14,107	18,490	32,597	0.65
East	South	10	9	578	12,837	5668	18,505	0.51
East	West	10	21	301	10,400	12,567	22,967	0.58
North	South	9	9	5269	22,669	11,215	33,884	0.69
North	West	9	21	4482	20,233	17,887	38,120	0.73
South	West	9	21	111	3842	13,177	17,019	0.41

**Table 5:** Pairwise comparison of private alleles and fixed differences between karyotypes. Karyotypes, the number of individuals per karyotype, the number of loci with fixed allele differences between each karyotype, the private alleles for each karyotype, the total private alleles for each pairwise comparison between karyotypes and the Fst between karyotypes

Karyo1	Karyo2	N1	N2	Fixed	priv1	priv2	Total priv	Fst
AA	MA	10	10	0	6041	6683	12,724	0.095
AA	MM	10	15	13	16,413	10,529	26,942	0.163
MA	MM	10	15	0	14,399	7873	22,272	0.103

**Fig. 3:**
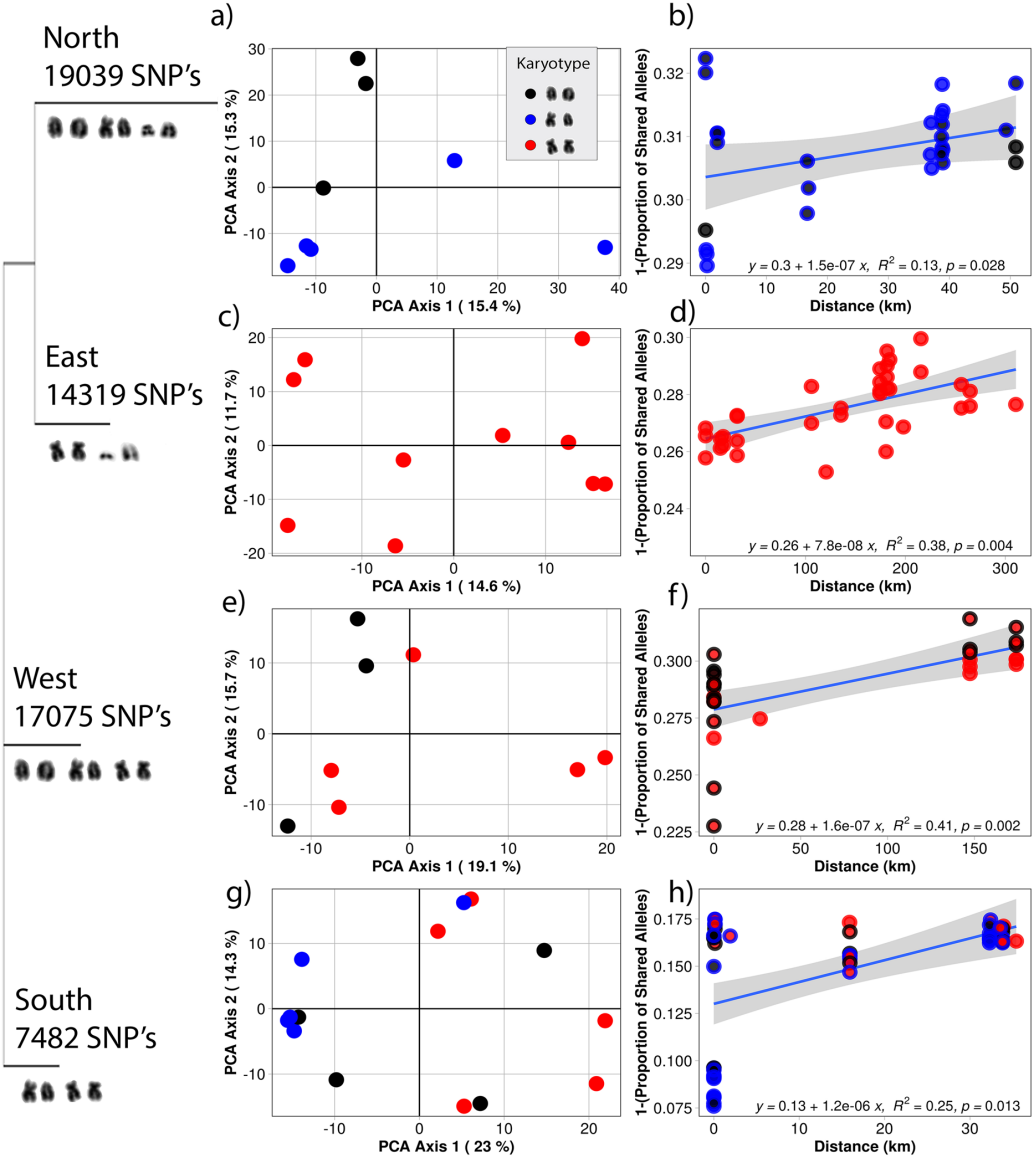
Within-population genetic distances for each population and karyotype identities for each individual, where homozygous acrocentric (AA) are black, heteromorphic (MA) are blue, and homozygous submetacentric (MM) are red. The relationship between each population is demonstrated with a neighbor-joining tree using the total SNPs for the metapopulation. The karyotypes for each population and the population specific SNPs are indicated on each node of the tree. **a** The north PCA and **b** IBD plots show that there was some structure associated with each karyotype, but we observed admixture between them within the population. **c** The east PCA and **d** IBD were fixed for submetacentric morphology and had the lowest genetic distances between individuals but the greatest collection distances. **e** The south PCA and IBD **f** had the fewest within-population SNPs and a high percentage of homozygous submetacentric individuals. An isolation-by-distance effect was observed, and there was evidence of admixture between heterokaryotypes. **g** The west PCA and **h** IBD had all three karyotypes and showed evidence of gene flow between each type. There is evidence of admixture in both the PCA and the isolation-by-distance plot. The west also had the highest within-population genetic distances and the second highest total SNPs

**Fig. 4:**
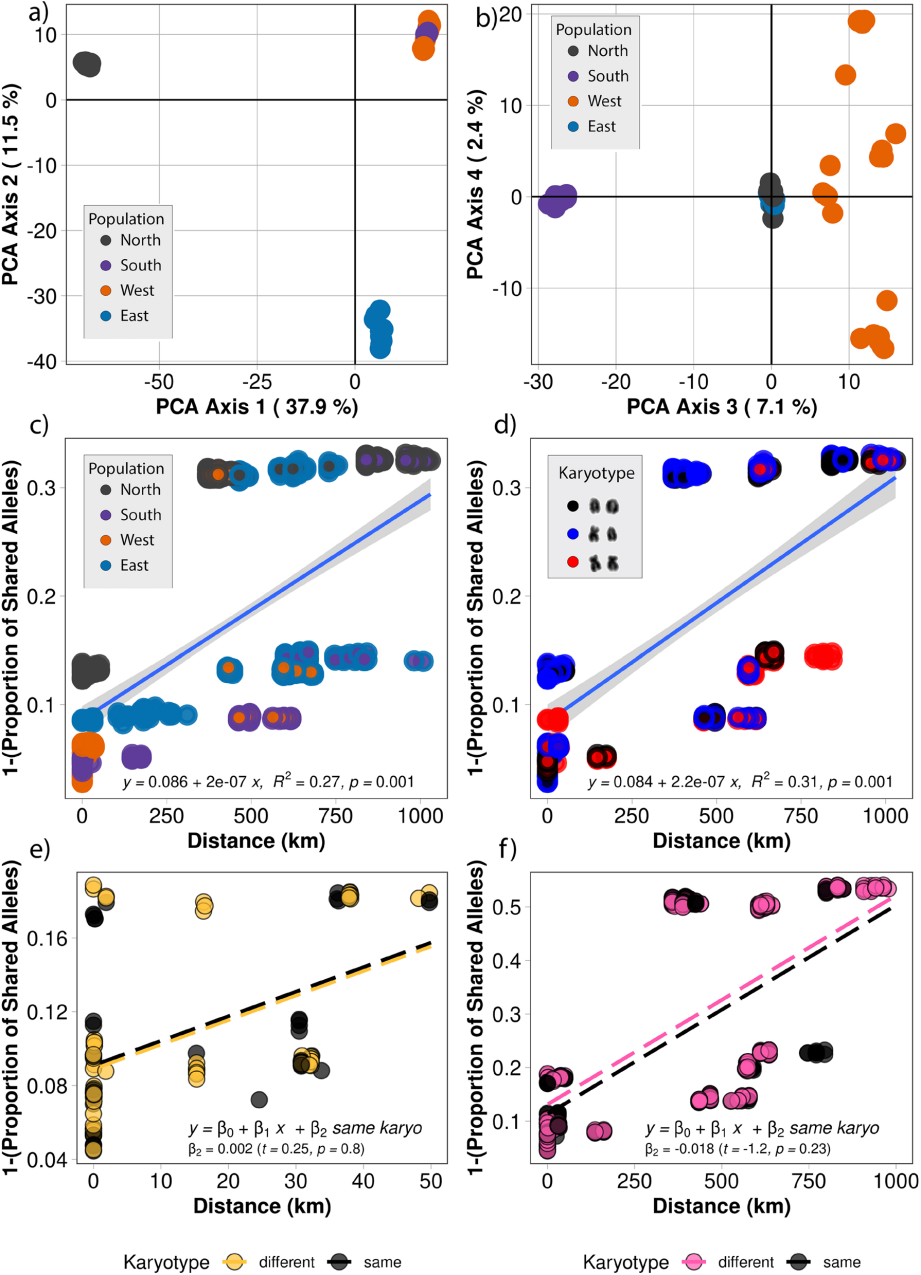
**a** and **b** PCA of populations based on the ordination of Euclidean distance from 46,189 SNPs. Axes 1 and 2 (**a**) show a clear separation of the north, east, and west/south populations, and axes 3 and 4 (**b**) show that the west and south are also distinct. **c** Isolation by distance based on mantel test for unshared alleles of individuals vs distance and **d** populations for *F*_st_ vs distance. **e** Multiple linear *regression* model *for within*-population (< 50 km) testing the same versus different karyotypes over using genetic distance and Euclidean distance. **f** Multiple linear *regression model for between* populations (< 1000 km) testing same versus different karyotypes over using genetic distance and Euclidean distance

We found no evidence that karyotype differences were genome-wide inhibitors of gene flow based on our multiple linear regression model (*t*_102_ = 0.63, *p* = 0.53) (Fig. 4e). To further analyze if the chromosome polymorphisms were barriers to gene flow within populations, we used both ordinations of allele frequency and distribution of karyotypes using PCA and isolation by distance for each population (Fig. 3). The data from the western population was the most informative because all three karyotype morphologies for chromosome 6 were present within a very short distance (< 1 km) and this population had the greatest genetic distances in the PCA (23% and 14.3% on axes 1 and 2). We did not observe any obvious population structure associated with karyotype differences within this population; instead, we observed a lack of structure between karyotypes in both the PCA and the isolation-by-distance plots (Fig. 3d). In the south (Fig. 3c), however, the north population (Fig. 3a) had admixture between heteromorphic and homomorphic karyotypes. The east had a fixed submetacentric chromosome 6 and also showed the lowest overall genetic distances in the PCA with 14.6% and 11.7% in the first and second axes (Fig. 3b). All populations showed an isolation-by-distance effect, but this effect was not associated with karyotype differences.

### Genetic structure between populations

The genetic structure between populations was evaluated using PCA, IBD, *F*_st_, and fixed differences. First, PCA analysis of the 46,189 SNP loci shared between all populations showed four distinct genotype clusters with no observable genetic admixture between the north, east, west, and south subpopulations representing 37.9%, 11.5%, 7.1%, and 2.5% of the total genetic variation on PCA axes 1, 2, 3, and 4, respectively (Fig. 4a and b). Following the PCA analysis, we performed an isolation-by-distance analysis based on the Mantel test using a dissimilar measure of unshared alleles (1, the proportion of shared alleles) for all genotyped individuals. This showed an isolation-by-distance effect (*y* = 0.075 + 1.7e-07*x*, *R*^2^ = 0.266, *p* = 0.001) (Fig. 4c).

Next, we grouped individuals by chromosome 6 polymorphisms for each of the three karyomorphs and compared unshared alleles between them (*y* = 0.084 + 2.2e-07, *R*^2^ = 0.314, *p* = 0.001) and all three karyotypes clustered together (Fig. 4e). This demonstrated that genetic isolation and unshared alleles (private alleles) were not associated with the allelic differences associated with karyotype morphology. Individuals with different chromosome 6 karyotypes were found in the same location and shared the same alleles within their localities. Karyotype morphologies demonstrated close genetic relationships of individuals with different karyotypes indicating there was not a genome-wide restriction of gene flow between individuals with different karyotypes. We generated a multiple linear regression model to test if there was a significant difference between the same and different karyotypes to determine if these had an impact on the isolation-by-distance observation and found there was no significant difference (*t*_102_ =  − 1.19, *p* = 0.24) (Fig. 4f).

To test whether gene flow was restricted between populations, we performed fixed allele differences between each population and compared these values with *F*_st_ and private alleles (Table 4). Fixed allele differences can be a strong indicator of lack of gene flow because for a given allele to reach fixation in a population the opposite allele cannot be present in that population sample. The independent population analysis revealed that the northern population was the most divergent and had the greatest number of SNPs (Table 3), the fewest monomorphic loci (26,504), and the greatest number of fixed differences pairwise between all other populations with 5269 fixed differences with the south (22,669 private alleles), 4482 fixed differences with the west (20,233 private alleles), and 4076 fixed differences with the east (18,490 private alleles) (Table 4). We then used fixed allele analysis to test for restricted gene flow between karyotypes (Table 5) and identified 13 fixed allele differences between the homozygous acrocentric (AA) and homozygous submetacentric (MM) karyotypes but no fixed allele differences between either of the fixed karyomorphs with the heterokaryotypes (MA).

The population to the west had the second highest number of SNPs (17,075), the second fewest number of monomorphic loci (29,114) and 301 fixed differences with the east (12,567 private alleles), and 111 fixed differences with the south (13,177 private alleles). The west also shared 4508 SNP loci with the east and 3891 loci with the south, indicating that the east and south populations have diverged from the west more recently than the north.

The south population was dominated by homozygous submetacentric karyotypes and had the fewest number of SNP loci (7733), the highest number of monomorphic loci (38,439), and the fewest private alleles in all pairwise comparisons to the other populations indicating a genetic bottleneck (Table 3 and Table 4). This drift was characterized by a transition from a polymorphic population with a high frequency of homozygous acrocentric individuals (west) and high levels of genetic diversity to a population with greatly reduced genetic diversity (fixation of 13,184 alleles that are shared with the west as SNPs) and a high frequency of homozygous submetacentric (MM) individuals but still polymorphic with the presence of some (MA) individuals.

The east was the only population that had a fixed submetacentric chromosome 6, and the shared SNP loci indicate that the east had also diverged from the west. The east also had enlarged unpaired microchromosomes, but due to the low sample sizes associated with the microchromosome polymorphisms, we did not integrate these with the SNP datasets. The east population had fewer SNPs than both the north and the west but nearly twice as many as the south. This could be a result from geneflux or mutation and subsequent increased recombination upon the fixation of the derived rearrangement (MM:6) (Fig. 3c and d, Table 3).

Allele shuffling and parallel divergence of thousands of loci were observed in all populations. Submetacentric chromosomes were associated with increased monomorphic loci and fewer polymorphic loci (drift), whereas acrocentric chromosomes were associated with fewer monomorphic loci and increased SNP loci. When we plotted the flow of private and fixed alleles between populations pairwise and with the metapopulation (Table 4), it was clear that the recruitment of these alleles was facilitated by an independent assortment of large subsets of alleles unique to each population, but also many alleles that are all shared within the metapopulation have been recruited independently by each population at different allele frequencies and driven to fixation independently as monomorphic loci.

## Discussion

In this study, we used a “chromosomics” approach integrating populations genetics data with molecular cytogenetics (Claussen 2005; Deakin et al. 2019; Deakin and Potter 2019; Liehr 2019) to test if SNP profiles could be informative for determining the population-level influences of gene flow impacted by chromosome polymorphisms both within and between populations of *Varanus acanthurus*. We investigated intraspecific chromosome rearrangements within four geographically distinct populations of *V. acanthurus* to determine if chromosome morphology was indicative of population structure, i.e., to determine if chromosomal rearrangements drive population divergence or result from it. We discovered that there was no obvious population structure associated with chromosome rearrangements; however, chromosome rearrangements were widespread prior to the genetic divergence of populations.

### Chromosome polymorphisms in *V. acanthurus*

Our intraspecific cytogenetic analysis in *V. acanthurus* revealed three different chromosome arrangements: (i) polymorphism in chromosome 6, described previously (King et al. 1982) (Fig. 2b); (ii) polymorphisms involving microchromosomes, described previously (Matsubara et al. 2014); and (iii) interpopulation size variations of the Z and W sex microchromosomes, not previously reported (Fig. 2c). Intraspecific polymorphisms have been rarely reported in wild vertebrate populations. The notable exceptions of polymorphisms occurring in small mammals (Patton 1969; Poorman et al. 1981; Lindholm et al. 2019) and birds (Lowther 1961; Thomas et al. 2008; Sun et al. 2018; Grogan et al. 2019) represent a dynamic state that could be ephemeral or could be maintained by balancing selection for considerable time (Dobigny et al. 2017; Damas et al. 2021).

We observed polymorphisms at chromosome 6 in all study populations except to the east of the Barkly Tablelands where all individuals analyzed cytogenetically had a fixed submetacentric karyotype (Fig. 1, Fig. 2, and Fig. 3) confirming the results of a previous study (King et al. 1982). This suggests that populations east of the Barkly Tablelands are genetically isolated and are either divergent or represent the ancestral karyomorph. However, the karyotype analysis alone did not identify any distinct population structure in the other populations besides the widespread presence of chromosome rearrangements. Rearrangements involving chromosome 6 have been well documented in Varanidae and have been classified as pericentric inversions or centromere relocation. The two fixed homozygous morphologies (fixed submetacentric and fixed acrocentric) have been used for phylogenetic analysis in the construction of seven clades from 37 species endemic to Africa, the Middle East, Asia, and Australia (King and King 1975; King et al. 1982; Matsubara et al. 2014; Srikulnath et al. 2013; Patawang et al. 2017; Rovatsos et al. 2019a, b; Iannucci et al. 2019; Augstenová et al. 2021). However, only *V. acanthurus* was characterized by both homozygous fixed karyotypes and polymorphic karyotypes (King et al. 1982), suggesting that they could be undergoing chromosomally driven speciation in real-time with the heteromorphic karyotypes representing a widespread hybrid lineage.

We also identified multiple microchromosome polymorphisms in two populations of *V. acanthurus* (Fig. 2a)*.* These microchromosome polymorphisms are unusual because a recent study suggests that microchromosomes are highly conserved across all lower vertebrates (Waters et al. 2021). However, the molecular mechanism behind the evolution of autosomal microchromosomes is yet unknown. In addition, we do not know any other species where stepwise variations in microchromosome rearrangements have occurred, where one population with homozygous enlarged microchromosomes, another with heteromorphism (enlarged and typical sized), and other populations with homozygous microchromosomes (Fig. 2a). This is a very similar pattern to what has been observed before in this species or group involving macrochromosomes, for example, chromosome 6 (Fig. 2a and b). Although enlarged microchromosomes were identified previously in *V. acanthurus*, they have not been observed in any other varanid species (Matsubara et al. 2014). However, in that study, the authors identified an enlarged submetacentric microchromosome 9 that was unpaired in both males and females. This suggests that microchromosome polymorphisms are more common in *V. acanthurus* than previously identified.

The third chromosome polymorphism we observed was interpopulation size variation of the sex chromosomes, and this is a novel discovery. So far, in all species of varanids karyotyped, the W chromosome was larger than the Z, with the W chromosome size varying between species from only slightly larger to almost double the size of the Z (King and King 1975; King et al. 1982; Matsubara et al. 2014; Srikulnath et al. 2013; Patawang et al. 2017; Rovatsos et al. 2019a, b; Iannucci et al. 2019; Augstenová et al. 2021). Varanids have also been proposed to have the oldest conserved sex chromosomes among vertebrates (Rovatsos et al. 2019a, b; Iannucci et al. 2019). This suggests varying stages of evolutionary differentiation due to varying rates of accumulation of repetitive sequences on the varanid W chromosomes. In this study, we detected intraspecific size differences of W chromosomes similar to that of interspecies size variation of other varanid species, including homomorphic sex chromosomes in the northern population. This W size variation is intriguing because the north population with homomorphic sex chromosomes displayed all types of autosomal polymorphisms (Fig. 1, Fig. 2, and Fig. 3) but did not have heteromorphic sex chromosomes which are common in other varanids. This provides evidence of independent evolutionary rates of the sex chromosomes and autosomes in different populations. This variation of the sex chromosomes could be an artifact of the extreme divergence of the north from the other populations, and if these sex chromosomes are incompatible with the more common heteromorphic sex chromosomes from other populations, they could represent a genetic barrier and help explain the extreme divergence of this population.

### Absence of gene flow between populations

To determine the level of genetic diversity among four geographically distinct but chromosomally polymorphic populations of *V. acanthurus*, we performed population genetic analysis using SNP markers generated using Diversity Arrays Technology (DArTseq) (Kilian et al. 2012). Our population genetics analysis indicated that all four populations were genetically distinct from each other and there was no evidence of gene flow between them (Fig. 4, Table 4). This suggests that these four geographically distinct populations likely represent four genetically distinct conservation units, similar to what was shown in the study of the Komodo dragon (*V. komodoensis*), where three genetically distinct conservation units were identified from the five main Indonesian islands (Iannucci et al. 2021). A recent phylogeographic study on *V. acanthurus* that utilized SNP markers, morphometric data and weather data, identified a need for taxonomic revisions and considered *V. acanthurus* a species complex with at least one undescribed species from the Cape Crawford area, which is sympatric with our north population (Pavón-vázquez et al. 2022). It is therefore of significant interest that here we have shown the population structure of these widespread dwarf goannas was essentially “islands” in various stages of divergence similar to the observations of other species of varanids on the many islands of Indonesia (Welton et al. 2014; Weijola et al. 2019) but without any obvious geographical barriers. That could indicate that genetic barriers have been established following divergence and those barriers are independent of the chromosome polymorphisms that occur within the populations.

### Lack of genome-wide inhibition of gene flow between karyotypes

Although the within-population structure analysis did not indicate an abrupt sink of gene flow, there were some fixed allele differences between the two homozygous karyomorphs that could indicate recombination suppression between the fixed AA and MM morphs in the western population. This would be similar to the observations between *Drosophila persimilis* and *D. pseudoobscura* in which restriction of gene flow is only isolated to the rearranged region of the chromosome polymorphism (Fuller et al. 2018). Future work will require aligning our SNP data to the newly sequenced *V. acanthurus* genome (Zhu et al. 2022) to determine the location of the fixed allele differences and determine if they are in linkage disequilibrium. Then to validate if those loci are part of the rearrangement, a FISH probe designed from the sequence data would provide evidence that those fixed alleles are correlated with suppressed recombination from the rearrangement.

In conclusion, cytogenetic only data was inconclusive for defining population structure; however, when combined with the molecular data, it clearly provided evidence that chromosome rearrangements were widespread prior to the genetic divergence of these populations. The population genetics data provided this additional resolution to define fine-scale population structure that could not be resolved with cytogenetics alone. However, combining both cytogenetic and molecular data sets enabled the reconstruction of the history of chromosome transitions within *V. acanthurus* which is likely a complex of multiple cryptic species.

## Data Availability

Dobry, Jason (2022), population cytogenetics of the spiny-tailed goanna, Dryad, Dataset, 10.5061/dryad.tb2rbp03n
